# Supplementary material for: Methods of Inactivation of Highly Pathogenic Viruses for Molecular, Serology or Vaccine Development Purposes
Source: Pathogens. 2022 Feb 19;11(2):271. doi: 10.3390/pathogens11020271 (PMC8879476; doi:10.3390/pathogens11020271)
Supplement: Supplementary file 1 [file pathogens-11-00271-s001.zip › pathogens-1589344-supplementary.pdf]

pathogens

MDPI

Table S1: Inactivating protocols with correlation to virus family.

	Togaviridae	*Flaviviridae*	*Filoviridae*	*Arenaviridae*	*Hantaviridae/ Phenuiviridae/ Nairoviridae/ Peribunyaviridae*	*Orthomyxoviridae*
HEAT	Highly variable: CHIKV 56 °C 120 min; RRV 56 °C 120 min; ONNV 56 °C 40 min; BFV 56 °C 30 min, [14,39]	56 °C, 30 min or 60 °C 3 min (Alkhurma); 56 °C, 30 min (YFHV) [15,37]	60 °C, 60 min + denaturing solution, (BSL-4 pathogen) [11] 0.1% SDS + 0.1% Tween-20 + 60 °C, 15 min (BSL-4 pathogen) [6,11,13,17]	60 °C, 60 min (LASV) [13] or in combination with a denaturizing solution [11]		55.6 °C, 30 min (H3N2,H7N3,H1N1 p) [49].
SOLVENTS Alcohol/ acetone					Methanol (absolute), 8 min; acetone/methanol (1:1) 10 min. (HTNV) *Hantaviridae* [29]	
IONIC DETERGENTS						
Sodium dodecyl sulfacte (SDS)			0.1% SDS + 0.1% Tween-20 + 60 °C, 15 min (BSL-4 pathogen) [6,11,13,17]			
NON-IONIC DETERGENTS						
Triton X-100			AVL + 0.1% Triton X-100, 20 min. [7]			1% Triton X-100, 1 h, room temp (H3N2,H7N3,H1N1 p) [49].
Tween-20 / Tween-80			0.1% SDS + 0.1% Tween-20 + 60 °C, 15 min (BSL-4 pathogen) [6,11,13,17]			
ALDEHYDES						
Formaldehyde (CH_2O)		Formalin 0.2%, 120 h, 32 °C (WNV) [89]<br><br>0.02% formaldehyde, pH 7.4, 22 °C, 120 min (DENV 2) [18]				0.04% formalin, 16 h, 37 °C; 0.02% formalin, 48 h 37 °C. (HPAI: H5N1; LPAI:: H9N2, H4N6, H11N1) [78]<br><br>0.02% formalin, 18 h, room temp (H3N2,H7N3,H1N1 p) [49].
Glutaraldehyde ($HCO\text{-}(CH_2)_3\text{-}CHO$)	0.1%, 24 h, 4 °C. (Sindbis v.) [82]					
Paraformaldehyde ($OH(CH_2O)_nH$)					1% paraformaldehyde, 20 min. (HTNV) *Hantaviridae* [29]	

GUANIDINE BASED CHAOTROPIC SALTS (nucleic acid extraction kits), e.g., Trizol LS, AVL buffer, Magna pure lysis/binding buffer (MPLB).	560 µL Trizol LS reagent, 10 min incubation, room temp.<br>(WEEV, EEEV and VEEV)<br>[2]	560 µL Trizol LS reagent, 10 min incubation, room temp.<br>(DENV, WNV)<br>[2]	560 µL Trizol LS reagent, 10 min incubation, room temp.<br>(EBOV, MARV)<br>[2,11]<br><br>AVL + 0.1% Triton X-100, 20 min [7]<br><br>MPLB directly in EDTA tube<br>(EBOV) [5]		560 µL Trizol LS reagent, 10 min incubation, room temp. (RVF) *Phenuiviridae*<br>[2]	
			PHOTOACTIVE COMPOUNDS			
1.5-iodonaphtyl azide (INA)	50 µM INA, UV-A 5 min. (CHIKV)<br>[82]	50 µM INA, UV-A 5 min (DENV 2) [18]	100 µM, 30 min, room temp + UV-A, 10 min (EBOV)<br>[80]			
Psoralen	AMT and UV-A light exposure (see figure 1 for dose)<br>(VEEV) [1]	AMT and UV-A light exposure (see Table 1 for dose)<br>(DENV, WNV, SLEV, YFHV) [1]<br><br>AMT 35 µM, UV-A 2 min [18]	AMT and UV-A light exposure (see Table 1 for dose)<br>(EBOV, MARV)<br>[1]	AMT and UV-A light exposure (see Table 1 for dose)<br>(LASV, JUNV) [1]	AMT and UV-A light exposure (see Table 1 for dose)<br>(CCHFV) *Nairoviridae,* (RVF) *Phenuiviridae* [1]	AMT and UV-A light exposure (see Table 1 for dose)<br>(H1N1p, H1N1, H3N2) [1]
Methylene blue (MB)		20 µM MB 20 min (in dark), 40 W fluorescent bulb, 10 min, 3log reduction (WNV)<br>[86]				
Riboflavin (vitamin B2)						
OXIDIZING AGENTS:						
Hydrogen peroxide (H_2O_2)		3% H_2O_2, 2 h, pH 7.4, 20–24 °C<br>(WNV, YFHV)<br>[58]		3% H_2O_2, 2 h, pH 7.4, 20–24 °C<br>(LCMV) [58]		1% H_2O_2, 2 h, room temp (H1N1, $H1N1_{pdm09}$, H3N2)<br>[59]
			ALKYLATING AGENTS			
Beta-propiolactone (BPL)		BPL 0.1%, 48 h, 4 °C (WNV) [89].				0.1% BPL, 16 h, 4 °C, hydrolysis 2 h, 37 °C (HPAI: H5N1; LPAI: H9N2, H4N6, H11N1) [78].<br><br>0.094% BPL, 16 h, 4 °C, hydrolysis 2 h, 37 °C<br>(H3N2,H7N3,H1N1p) [49].

						0.02% BPL, 24 h, 4 °C, hydrolysis 7 h, 37 °C (H3N2) [73].
IRRADIATION						
UV-radiation	6000 μJ/cm[2] of UVC; 40,000 μJ/cm[2] of UVC in microcrystalline form (Sindbis v.) [82]				UV-A (312 nm), 3 min. (HTNV) *Hantaviridae* [29]	
Gamma-irradiation			3Mrad gamma (rVSV encoding EBOV-gp) [45]		3Mrad gamma (LACV) *Peribunyaviridae* [45]	
DISULFIDE BASED COMPOUNDS				40 μM NSC20625, 90 min, 37 °C (JUNV) [51,52].		
